# OTUD4 promotes the progression of glioblastoma by deubiquitinating CDK1 and activating MAPK signaling pathway

**DOI:** 10.1038/s41419-024-06569-x

**Published:** 2024-03-01

**Authors:** Mingxin Ci, Gaichao Zhao, Chongyang Li, Ruochen Liu, Xiaosong Hu, Jun Pan, Yang Shen, Guanghui Zhang, Yongsen Li, Li Zhang, Ping Liang, Hongjuan Cui

**Affiliations:** 1 State Key Laboratory of Resource Insects, Medical Research Institute, Southwest University, Chongqing, 400715 China; 2 Jinfeng Laboratory, Chongqing, 401329 China; 3 Department of Radiology and Nuclear Medicine, The First Hospital of HeBei Medical University, Hebei, 050000 China; 4 Department of Neurosurgery, Children’s Hospital of Chongqing Medical University, National Clinical Research Center for Child Health and Disorders, Ministry of Education Key Laboratory of Child Development and Disorders, Chongqing, 400014 China

**Keywords:** Cancer, Cell biology

## Abstract

Glioblastoma, IDH-Wild type (GBM, CNS WHO Grade 4) is a highly heterogeneous and aggressive primary malignant brain tumor with high morbidity, high mortality, and poor patient prognosis. The global burden of GBM is increasing notably due to limited treatment options, drug delivery problems, and the lack of characteristic molecular targets. OTU deubiquitinase 4 (OTUD4) is a potential predictive factor for several cancers such as breast cancer, liver cancer, and lung cancer. However, its function in GBM remains unknown. In this study, we found that high expression of OTUD4 is positively associated with poor prognosis in GBM patients. Moreover, we provided in vitro and in vivo evidence that OTUD4 promotes the proliferation and invasion of GBM cells. Mechanism studies showed that, on the one hand, OTUD4 directly interacts with cyclin-dependent kinase 1 (CDK1) and stabilizes CDK1 by removing its K11, K29, and K33-linked polyubiquitination. On the other hand, OTUD4 binds to fibroblast growth factor receptor 1 (FGFR1) and reduces FGFR1’s K6 and K27-linked polyubiquitination, thereby indirectly stabilizing CDK1, ultimately influencing the activation of the downstream MAPK signaling pathway. Collectively, our results revealed that OTUD4 promotes GBM progression via OTUD4-CDK1-MAPK axis, and may be a prospective therapeutic target for GBM treatment.

## Introduction

Glioblastoma, IDH-Wild type(GBM, CNS WHO Grade 4 [1–3]) is a highly heterogeneous and aggressive primary malignant brain tumor with a median survival of 14 months [4, 5]. Despite improved surgical strategies and vigorous radiation and chemotherapy [6], the prognosis for GBM patients is poor [7]. It is urgent to find new drug targets for the treatment of GBM.

OTU deubiquitinase 4 (OTUD4) is a deubiquitinase that belongs to the Ovarian tumor-associated proteases domain-containing proteins (OTUDs) family. Research confirmed that OTUD4 is a potential predictor of several human cancers [8] and is also involved in DNA alkylation damage repair, which is important in cancer radiation and chemotherapy [9, 10]. However, the biological function of OTUD4 in GBM has not been elucidated.

Here, we report that OTUD4 is significantly overexpressed in glioblastoma and is important for cell proliferation, invasion, and clonogenic capacity. Mechanistically, on the one hand, we demonstrated a direct protein interaction between OTUD4 (181-300aa) and CDK1, and OTUD4 regulates the stability of CDK1 by deubiquitination. OTUD4, on the other hand, indirectly stabilizes CDK1 by binding to and deubiquitinating FGFR1, which then positively affects the MAPK pathway. In conclusion, our findings demonstrated that the OTUD4-CDK1-MAPK axis is critical for promoting GBM progression. Our study, therefore, may offer insights for finding novel, feasible glioblastoma targets and new anticancer therapies.

## Materials and methods

### Cell culture

Human glioblastoma cell lines (A172, LN229, U87MG, and U118MG), human astroglia cells SVGP12, and human embryonic kidney (HEK) 293FT cells were originally obtained from American Type Culture Collection (ATCC, Beijing, China). All cell lines were tested mycoplasma-negative [11]. Cell lines were cultured in Dulbecco’s Modified Eagle’s Medium (DMEM) (Gibco: Grand Island, NY, USA) supplemented with 10% (vol/vol) fetal bovine serum (Vivacell: Chongqing, China) at 37 °C, in 5% CO_2_ humid atmosphere.

### Reagents and antibodies

Cycloheximide (CHX) (Merck: Kenilworth, NJ, USA, CAS: 66-81-9), MG132 (MCE: Chongqing, China, CAS:133407-82-6). The primary antibodies used are described in Table 1.

**Table 1:** Antibodies used in the experiment.

Antibody	Catalog No.	Corporation	Address
OTUD4	25070-1-AP	Proteintech	Wuhan, China
CDK1	67575-1-lg		
MYC-tag	16286-1-AP		
Flag-tag	66008-4-Ig		
CyclinB1	28603-1-AP		
p21	10355-1-AP		
p27	25614-1-AP		
MMP9	10375-2-AP		
ERK1/2	11257-1-AP		
p-ERK1/2 (Thr202/Tyr204)	28733-1-AP		
α-Tubulin	11224-1-AP		
E-cadherin	20874-1-AP		
N-cadherin	22018-1-AP		
HA-tag	13246	Cell Signaling Technology	Shanghai, China
FGFR1(for IHC)	9740S		
FGFR1(for western blot)	ab76464	Abcam	
BRAF	ET1608-36	HUABIO	Hangzhou, China
p-BRAF(T401)	ET1701-20		
MEK1/2	ET1602-3		
p-MEK1 (S218/S222)	ET1609-50		
Ki67	HA721115		
p-BRAF(T401)	bs-5224R	BIOSS	Beijing, China

### Plasmids, transfection, and infection

For OTUD4 and FGFR1 reduction, short hairpin RNA (shRNA) sequences were synthesized by Sangon biotech (Shanghai, China) and were inserted into the pLKO.1-puro vector (Table 2). Flag-tagged OTUD4 were subcloned into the pCDH-CMV-MCS-EF1-GFP-Puro vector (Youbio, Hunan, China). The recombinant plasmids expressing HA-tagged wild-type ubiquitin were purchased from Addgene (Beijing, China). HA-tagged-ubiquitin mutant plasmids (K6R, K11R, K27R, K29R, K33R, K48R, K63R) were purchased from Unibio Biotech (Changsha, China). Plasmids encoding MYC-tagged CDK1 mutants were obtained from GeneCreate Biotech (Wuhan, China).

**Table 2:** Sequences for shRNA.

Gene	Sequence
shOTUD4#1	CACTATAGATTCCAAACATAA
shOTUD4#2	GATATTGTGTATCCCATAAAG
shFGFR1#1	CCACAGAATTGGAGGCTACAA
shFGFR1#2	TGCCACCTGGAGCATCATAAT

The transfection and infection experiments were performed as described previously [12].

### Western blot analysis

For western blot analysis, cell lysates were prepared in RIPA lysis buffer containing protease inhibitors and phosphatase inhibitors. Proteins of different molecular weights were separated by SDS/PAGE and then transferred onto a polyvinylidene difluoride membrane, which was blocked with BSA or nonfat milk for 2 h. The membrane was then sequentially incubated with specific primary antibodies and secondary antibodies. We finally visualized it using the ECL Prime western blot detection system (Thermo Fisher, Shanghai, China).

### Proximity ligation assay (PLA)

Proximity ligation assay was carried out using Duolink® In Situ Red Starter Kit (#DUO92101, Sigma-Aldrich) as described previously [13].

### Quantitative real-time PCR (qRT-PCR)

Total cellular RNA was extracted using TriZol and cDNA was prepared by the Reverse Transcription Kit (Promega). SYBR qPCR SuperMix Plus (Novo Protein: Shanghai, China) was used for qRT-PCR analyses. Expression levels of the target genes were calculated according to the comparative Ct method (∆∆CT) (Table 3).

**Table 3:** QRT-PCR primer sequences.

Gene	Primer sequence
OTUD4-forward (5′-3′)	TTCTGATGTGGATTACAGAGGGC
OTUD4-reverse (5′-3′)	ACGCATGTTGTCTTACTCCTGA
CDK1-forward (5′-3′)	AAACTACAGGTCAAGTGGTAGCC
CDK1-reverse (5′-3′)	TCCTGCATAAGCACATCCTGA
FGFR1-forward (5′-3′)	CCCGTAGCTCCATATTGGACA
FGFR1-reverse (5′-3′)	TTTGCCATTTTTCAACCAGCG
GAPDH-forward (5′-3′)	GGAGCGAGATCCCTCCAAAAT
GAPDH-reverse (5′-3′)	GGCTGTTGTCATACTTCTCATGG

### MTT assay

A total of 1 × 10^3^ cells per well were seeded into a 96-well plate and cultured at 37 °C. 2-(4,5-dimethyltriazol-2-yl)-2,5-diphenyl tetrazolium bromide (MTT, Sigma) was added and incubated for 2 h, then cultured with 200 µL DMSO per well. Absorbance at a wavelength of 560 nm was measured by a microplate reader to detect the cell proliferation curves. Repeat the steps for 7 days.

### Transwell assay

A total of 5 × 10^4^ cells per well were seeded into the upper chamber (Corning, Beijing, China, pore size 8-µm) with 200 µL serum-free medium. For invasion experiments, Matrigel (BD Biosciences) was additionally added to coat the membrane. About 500 µL medium containing 10% FBS was added below the chamber. After approximately 8–10 h, the migration and invasion cells were fixed with paraformaldehyde solution, stained with crystal violet, and examined for numbers by microscopy after wiping.

### Colony formation assay

A total of 1 × 10^3^ cells per well were seeded into a 6-well plate and cultured at 37 °C for 7 days. The colonies were fixed with 4% paraformaldehyde, stained with crystal violet, and scanned under a scanner.

### Cell cycle analysis

Collected and resuspended cells in 75% ethanol at 4 °C overnight, then centrifugated and washed them with 1× PBS. Finally, stained cells with RNase A (100 µg/ml) and propidium iodide (PI, 50 µg/ml) for 30 min at 37 °C in the dark, then analyzed by flow cytometry (BD Biosciences, San Jose, CA, USA).

### Ubiquitination and protein half-life assay

For ubiquitination detection, the required plasmids, such as Flag-OTUD4, MYC-CDK1, and HA-UB were co-transfected into 293FT cells. Cells were treated with the proteasome inhibitor MG132 (10 µM) for 6–8 h before collection and then used for the evaluation of the ubiquitination levels of CDK1 or FGFR1. For the protein half-life assay, cells were treated with the protein synthesis inhibitor cycloheximide (CHX), after which cells were collected in a temporal gradient for the indicated durations. Protein levels were analyzed by western blot analysis and ImageJ.

### Xenograft studies

Animal experiments were approved by the Institutional Animal Care and Use Committee of Southwest University (IACUC-20221028-01) and were conducted strictly with the “Guidelines for Animal Care and Use” (Ministry of Science and Technology of China, 2006). Four-week-old female nude mice (BALB/c-nu) were purchased from Hunan SJA Laboratory Animal Co., Ltd (Hunan, China), housed in an SPF chamber with constant temperature and humidity, and were randomly assigned into two groups (12/group). The mice were anesthetized before injection to reduce pain. Then 1 × 10^5^ human GBM cells stably transfected with shGFP and shOTUD4 were collected, resuspended in 6 µl PBS, and injected into the brain of each mouse. After that, mice were sterilized with 75% medical alcohol. According to literatures and previous experimental experience, the mice were killed by cervical dislocation after about 4–6 weeks, and the brains were immersed in 4% paraformaldehyde. Randomization and single blinding were used for following experiments.

### Immunohistochemistry staining (IHC)

The samples were dehydrated, paraffin-embedded, cut into 5–10-μm-thick cross sections, which were sequentially dewaxed, rehydrated, antigen repaired, and inactivated endogenous peroxidase and organisms. After being sealed with goat serum and incubated with antibodies overnight at 4 °C, a reaction enhancer and an enhanced enzyme-labeled goat anti-rabbit/mouse IgG polymer were added. The sections were cleaned with PBS between each step. Finally, the DAB system was used to amplify the detection signal, and hematoxylin was used for re-staining, then dehydrated and sealed and observed under a microscope [14]. IHC assessment was produced by two independent observers (and a third in the case of strong disagreement) in a blinded fashion (i.e., no prior knowledge of clinical picture and patient outcome) [15].

### Statistical analysis

Statistics in the experiments were all performed by GraphPad Prism 7.0. All data in this study were analyzed and presented as mean ± standard deviation (SD) of at least three independent experiments. The two-tailed unpaired Student’s t-test was used to compare the results differences between groups. If the *p*-value < 0.05, the data were confirmed to be significant; *p < 0.05, **p < 0.01, ***p < 0.001.

## Results

### OTUD4 is upregulated in human GBM and positively associated with poor prognosis

**Fig. 1:**
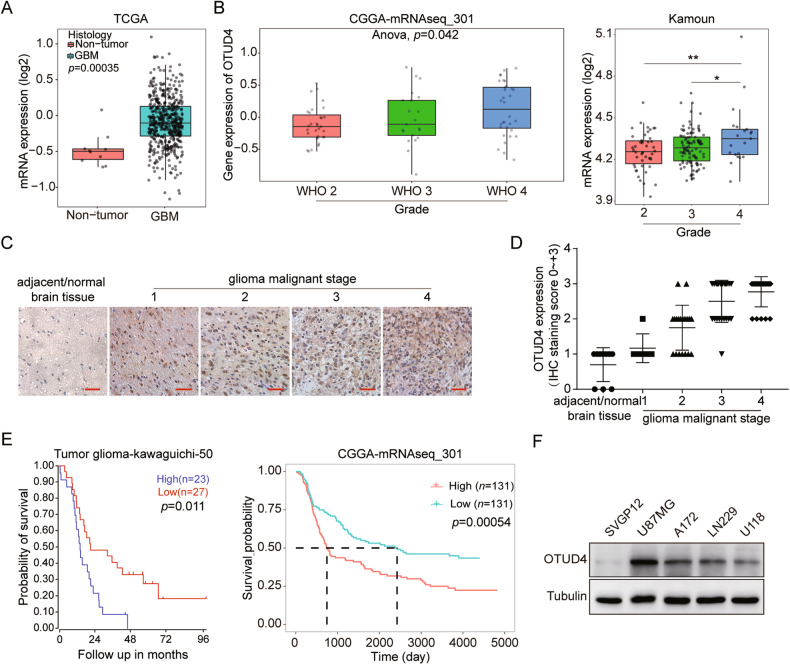
Abnormal activation of OTUD4 is positively correlated with poor GBM patient’s prognosis. **A** Box plot of OTUD4 expression levels in human GBM and adjacent normal brain tissues. **B** Box plots of OTUD4 expression levels in Grade glioma set. **C** Representative immunohistochemical staining of OTUD4 expression in different levels of human glioma and near-normal brain tissue. Scale bar, 100 μm. **D** Immunohistochemical scores of the expression level of OTUD4 in all tissue samples. **E** Kaplan–Meier analysis of overall survival probability using R2 and CGGA databases. **F** Western blot assay was used to observe the protein expression of OTUD4 in five cell lines including SGVP12, U87 MG, A172, LN229, and U118 MG.

To explore the expression of OTUD4 in glioblastoma, we analyzed the TCGA, CGGA, and GlioVis databases and found that compared with matched adjacent normal tissues, the expression level of OTUD4 in glioblastoma tissues was higher (Fig. 1A), and OTUD4 expression increased with glioma clinical grade deepened (Fig. 1B). Meanwhile, OTUD4 has the highest expression in GBM (the top level of glioma), and the increase of OTUD4 gene copy number was significantly correlated with its expression level in glioma (Supplementary Fig. 1A, B). The Human Protein Atlas database also revealed that the positive rate of OTUD4 in glioblastoma was significantly higher than that in normal human tissues (Supplementary Fig. 1C). Consistent with the database results, immunohistochemical staining on tissue samples from glioma patients showed that the expression level of OTUD4 was significantly increased in glioma, especially in grade 4 tissues (Fig. 1C, D). Then, we detected that abnormal activation of OTUD4 expression was positively correlated with poor prognosis in GBM patients (Fig. 1E). Additionally, western blot analysis showed that OTUD4 protein expression in four GBM cell lines was higher than that in astrocytes SVGP12 (Fig. 1F). Taken together, these results indicated that OTUD4 is overexpressed in GBM, and OTUD4 might be a potential predictor of poor prognosis in GBM.

### OTUD4 promotes GBM cell proliferation and invasion

**Fig. 2:**
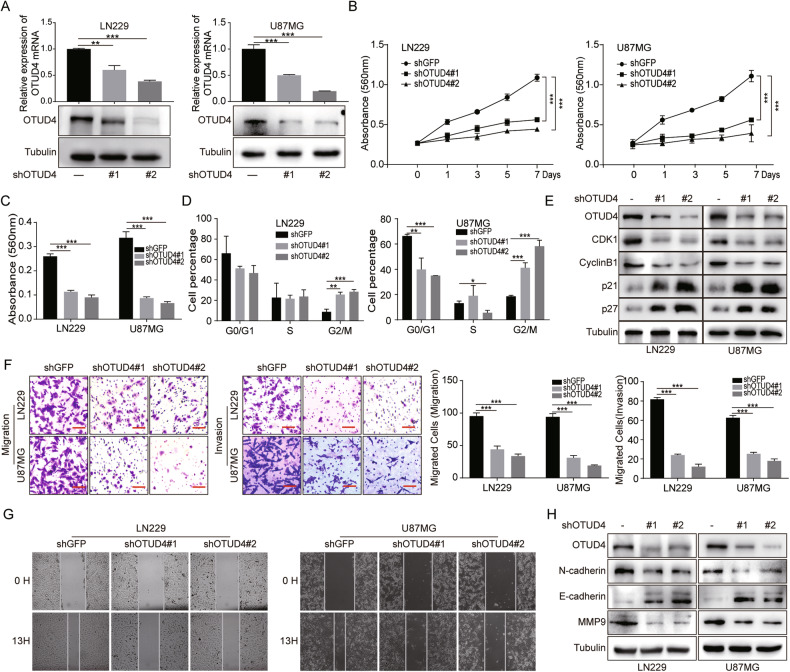
OTUD4 positively regulates cell proliferation and invasion of GBM cells. **A** Western blot and qRT-PCR assays were adopted to detect the expression levels of OTUD4 in the control and OTUD4-knockdown LN229 and U87MG cells. **B**, **C** MTT assays and plate cloning assays were performed to test the effect of OTUD4 knockdown on cell proliferation. **D**, **E** The cell cycle distribution was analyzed in LN229 and U87MG cells by flow cytometry, and western blot assay were used to detect G2/M cell cycle-related proteins. **F** Transwell assays were carried out in the control and OTUD4-knockdown GBM cells to detect invasion ability. Scale bar, 20 μm. **G** Wound-healing assay was performed in the control and OTUD4-knockdown cells. **H** Western blot assay was conducted to detect the expression levels of invasion marker proteins. All data were expressed as the mean ± SD, *n* = 3. Student’s t-test was performed to analyze significance. *P < 0.05, **P < 0.01, ***P < 0.001.

To further confirm whether OTUD4 affects GBM progression, stable OTUD4 downregulated GBM cell lines were constructed (Fig. 2A). OTUD4-knockdown GBM cells showed a dramatic decrease in cell number, accompanied by significant morphological changes (Supplementary Fig. 2A). Next, MTT assay showed that OTUD4 knockdown restrained the growth rate of GBM cells (Fig. 2B). Meanwhile, the results of plate cloning assay also revealed that the colony forming ability of GBM cells was decreased after OTUD4 knockdown (Fig. 2C, Supplementary Fig. 2B). Then, flow cytometry experiment observed that OTUD4 knockdown induced cell cycle arrest mainly in the G2/M phase (Fig. 2D, Supplementary Fig. 2C). Western blot consistently confirmed that the expression of G2/M-phase signature proteins CDK1 and cyclinB1 reduced, p21 and p27 upregulated (Fig. 2E). Next, transwell assay and wound-healing assay results indicated that the invasion capacity of GBM cells were markedly decreased after OTUD4 knockdown (Fig. 2F, G, Supplementary Fig. 3A). Subsequently, we examined marker proteins of invasion, the protein expression levels of N-cadherin and MMP9 were notably reduced, while E-cadherin expression was increased (Fig. 2H).

To further avoid the mistarget effect, recovery assays were performed and indicated that OTUD4 overexpression could partially rescue the proliferation and invasion capacity of GBM cells with OTUD4 knockdown (Supplementary Fig. 2D–F, Supplementary Fig. 3B–D). In addition, overexpression of OTUD4 notably promoted the proliferation and invasion of LN229 cells (Supplementary Fig. 2G–I, Supplementary Fig. 3E–G). Overall, our results concluded that OTUD4 is a key regulator of the proliferation and invasion of GBM cells.

### The 181-300aa of OTUD4 interacts with CDK1

**Fig. 3:**
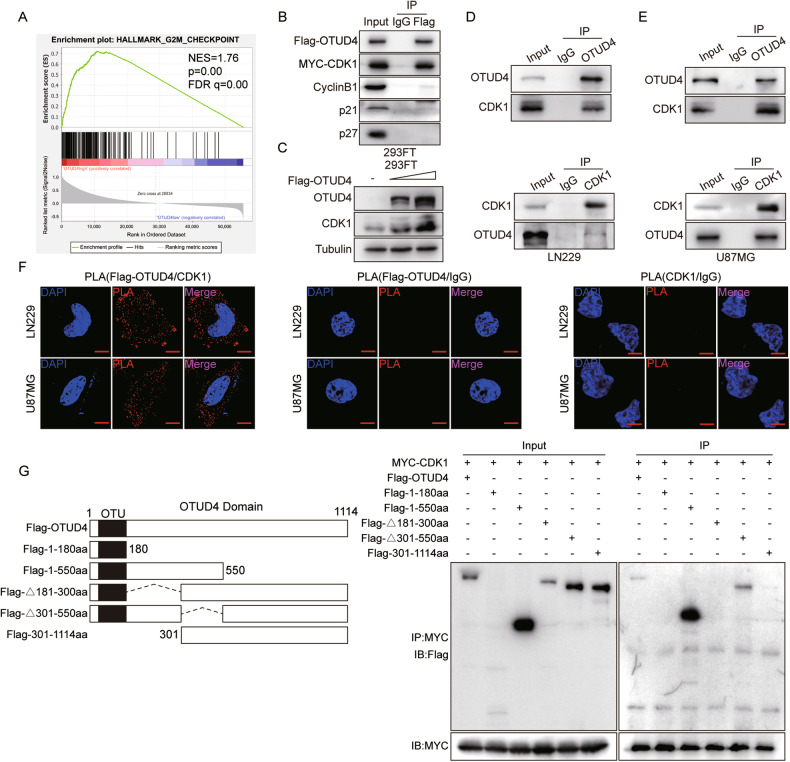
The 181–300aa of OTUD4 interacts with CDK1. **A** GSEA plots depicted enrichment of the cell cycle G2/M checkpoint in the samples with high OTUD4 expression. FDR, false discovery rate; NES, normalized enrichment score. **B** Co-IP assay were performed to detect interaction between OTUD4 and G2/M cell cycle-related proteins in 293FT cells. **C** The influence of gradient ectopic expression of OTUD4 on CDK1 was detected by western blot assay. **D**, **E** Co-immunoprecipitation assays were performed to detect interaction between OTUD4 and CDK1 proteins in LN229 and U87MG cells. **F** The proximity ligation (PLA) assay was applied to detect the interaction of Flag-OTUD4 and CDK1/IgG, CDK1 and IgG in GBM cells. Scale bar, 10 μm. **G** Interaction detection between CDK1 and OTUD4 truncated domains in 293FT cells.

We next explored the mechanism of OTUD4 in glioblastoma. We used the CGGA database for GSEA analysis, and the results showed that the high expression of OTUD4 was significantly positively correlated with the mitotic and cell cycle G2/M checkpoint in GBM (Fig. 3A, Supplementary Fig. 4A). Western blot analysis in the previous period also verified that, after OTUD4 was knocked down or overexpressed, the expression levels of CDK1, cyclinB1, p21 and p27 changed correspondingly (Fig. 2E, Supplementary Fig. 2F, I). However, further co-IP experiments showed that only CDK1 has an obvious and strong interaction with OTUD4 (Fig. 3B). And considering that CDK1 is an important regulator of cell proliferation and invasion [16], it is highly upregulated in GBM and is positively associated with poor patient prognosis (Supplementary Fig. 4B–D), we ultimately excluded other genes and selected CDK1 as a potential target gene of OTUD4 for follow-up experiments. Previous experiments discovered that the cell cycle was arrested in the G2/M phase after OTUD4 depletion, and the expression of CDK1 was markedly downregulated, but there was no significant difference in CDK1 mRNA levels (Supplementary Fig. 4E), indicating that the downregulation of CDK1 may occur at the post-transcriptional level. Ubiquitin-proteasome protein degradation pathway plays a key role in regulating protein levels. Interestingly, OTUD4 is a deubiquitylase. As expected, ectopic expression of OTUD4 resulted in CDK1 elevation in a dose-dependent manner (Fig. 3C). Therefore, we speculated that OTUD4 may interact with CDK1 to deubiquitinate and stabilize it.

Next, binding assays revealed that endogenous expression OTUD4 and CDK1 have physical interaction (Fig. 3D, E). Moreover, the proximity ligation assay (PLA) assay also corroborated a direct in situ interaction of OTUD4 with CDK1(Fig. 3F). To further validate their interaction regions, we constructed a set of Flag-tagged plasmids expressing different OTUD4 truncated mutants (full-length 1–1114aa, 1–180aa, 1–550aa, Δ181–300aa, Δ301–550aa, and 301–1114aa), and MYC-tagged CDK1 deletion mutant plasmids (N-terminal deletion of 1–99aa, intermediate deletion of 100–198aa, C-terminal deletion of 199–297aa) for transfection experiments. Results demonstrated that the 181–300aa domain of OTUD4 (Fig. 3G) and the full-length of CDK1 (1–297aa) (Supplementary Fig. 4F) mediated their physical interaction. This result is also consistent with our simulated molecular docking results of HADDOCK database [17–21] (Supplementary Fig. 4G).

### OTUD4 maintains CDK1 stability through deubiquitination

**Fig. 4:**
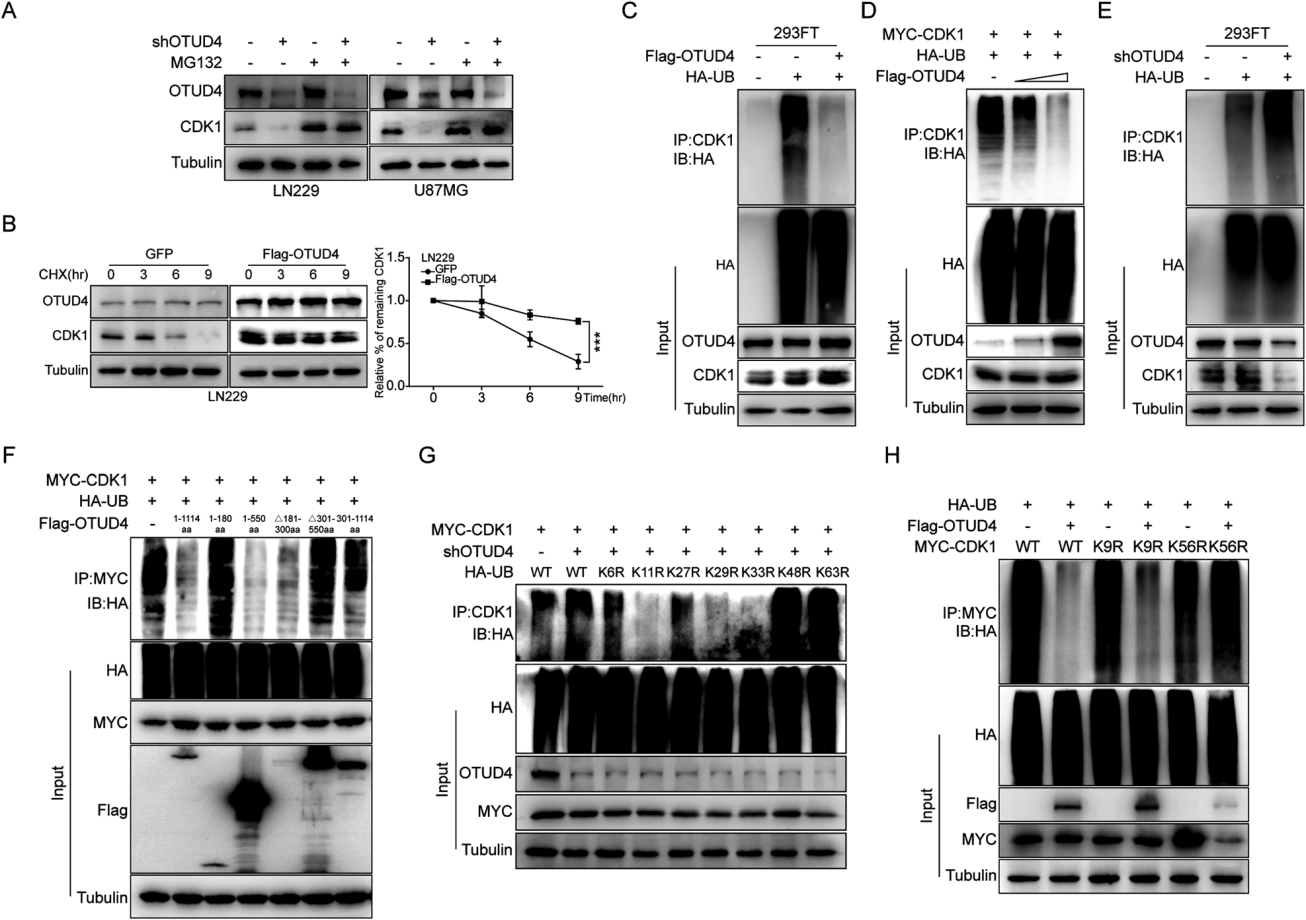
OTUD4 stabilizes CDK1 by removing the Lys 11, Lys 29 and Lys 33 types of ubiquitin chain on CDK1. **A** Western blot analysis of control and OTUD4-knockdown LN229 and U87MG cells treated with MG132. **B** Western blot analysis of the turnover of CDK1. **C–E** In the presence of MG132, 293FT cells were co-transfected with Flag-OTUD4 or shOTUD4, MYC-CDK1, and HA-UB plasmids, then the ubiquitination level of CDK1 was detected by co-IP. **F** In the presence of MG132, the MYC-CDK1, Flag-OTUD4 (deletion mutants), and HA-UB were co-transfected into 293FT cells for ubiquitination assays. **G** In the presence of MG132, the shOTUD4, MYC-CDK1, HA-UB, and ubiquitin mutant plasmids (only one lysine residue was mutated to an arginine residue) were co-transfected into 293FT cells for ubiquitination assays. **H** In the presence of MG132, the Flag-OTUD4, MYC-CDK1(wild-type and single-point mutants K9R, K56R) and HA-UB were co-transfected into 293FT cells for ubiquitination assays.

Next, we aimed to verify the possibility of the deubiquitination effect by OTUD4 on CDK1. MG132 (an inhibitor of the ubiquitin-proteasome pathway) dramatically blocks the downregulation of CDK1 caused by OTUD4 knockdown (Fig. 4A), suggesting that OTUD4 protects CDK1 from proteasome-dependent degradation. Then, cycloheximide (CHX, an inhibitor of protein synthesis) assay showed that the half-life of CDK1 was largely prolonged after OTUD4 overexpression, suggesting that OTUD4 specifically stabilizes CDK1(Fig. 4B, Supplementary Fig. 5A). Moreover, ubiquitination assays showed that the ubiquitination level of CDK1 could dramatically decrease after OTUD4 ectopic expression, and the decrease was concentration gradient dependent (Fig. 4C, D). In contrast, the downregulation of OTUD4 increased CDK1 polyubiquitylation level (Fig. 4E, Supplementary Fig. 6I). In conclusion, these results confirmed our speculation that OTUD4 directly stabilizes CDK1 by deubiquitination. Meanwhile, further assays showed that only when the full-length or 1–550aa of OTUD4 was overexpressed, the ubiquitination level of CDK1 was significantly reduced. And when 181–300aa was deleted or 301–1114aa was overexpressed, the ubiquitination level of CDK1 was partially reduced (Fig. 4F). Based on these, we speculated that 301–550aa of OTUD4 is the catalytic domain for deubiquitination of CDK1, and 181–300aa of OTUD4 might promote the DUB activity of OTUD4 on CDK1 by enhancing their interaction ability.

On the one hand, in order to identify which type of polyubiquitin modifications on CDK1 was affected by OTUD4, we performed a thorough deubiquitylation assay. The results suggested that downregulation of OTUD4 specifically enhanced wild-type and other ubiquitin mutants-linked polyubiquitylation of CDK1, except for K11R, K29R, and K33R (Fig. 4G). It was consistent with the results of ubiquitination experiments when OTUD4 is overexpressed (Supplementary Fig. 5B). On the other hand, to identify the possible key amino acid residues of CDK1 in this progression, we constructed two MYC-tagged CDK1 mutants with a single-point mutation (K9R, K56R) according to the PLMD (Protein Lysine Modifications Database) and UbiNet databases (Supplementary Fig. 5C). Co-IP results indicated that K56 residue of CDK1 is essential for OTUD4 deubiquitination (Fig. 4H).

### OTUD4 interacts with FGFR1 and stabilizes FGFR1 by deubiquitination

**Fig. 5:**
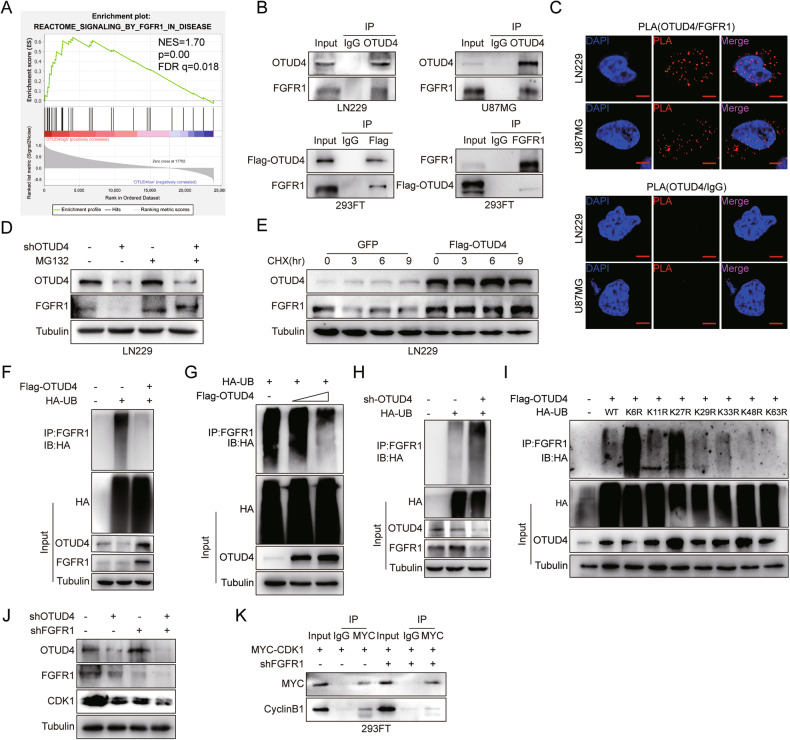
OTUD4 interacts with FGFR1 and stabilizes FGFR1 by deubiquitination. **A** GSEA plot depicted enrichment of the signaling by FGFR1 in disease in the samples with high OTUD4 expression. FDR, false discovery rate; NES, normalized enrichment score. **B**, **C** Co-immunoprecipitation assays and the proximity ligation (PLA) assay were performed to detect interaction between OTUD4 and FGFR1. Scale bar, 10 μm. **D** Western blot analysis of control and OTUD4-knockdown LN229 cells treated with MG132. **E** Western blot analysis of the turnover of FGFR1. **F–I** In the presence of MG132, 293FT cells were co-transfected with Flag-OTUD4 or shOTUD4, and HA-ubiquitin (wild type and mutants) plasmids, and the ubiquitination level of FGFR1 was detected by co-IP. **J** The effects of OTUD4 knockdown and FGFR1 knockdown on CDK1 protein levels were detected. **K** MYC-CDK1 and shFGFR1 were co-transfected into 293FT cells, and the interaction between CDK1 and cyclinB1 was detected by co-IP.

**Fig. 6:**
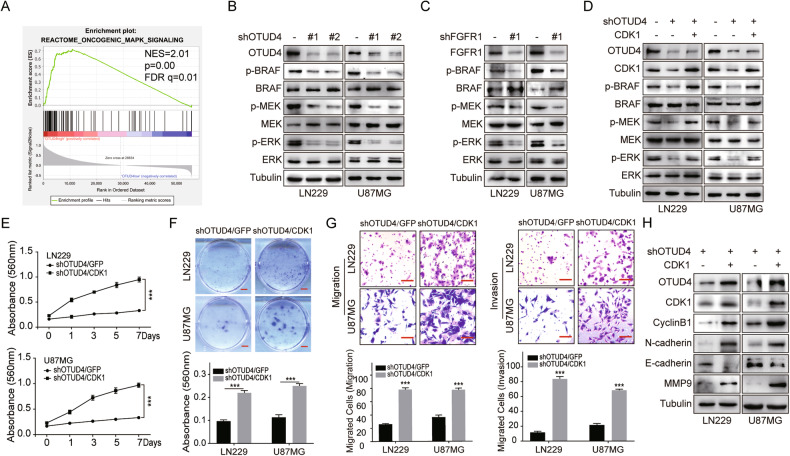
OTUD4 regulates GBM progression in a CDK1-dependent manner. **A** GSEA plot depicted enrichment of the MAPK pathway in the samples with high OTUD4 expression. FDR, false discovery rate; NES, normalized enrichment score. **B–D** Western blot was applied to detect phosphorylated proteins in the MAPK signaling pathway. **E**, **F** MTT and colony formation assays were used to examine proliferation ability of GBM cells. Scale bar, 2 mm. **G** Transwell assay was conducted to observe invasion ability of GBM cells. Scale bar, 20 μm. **H** Western blot was applied to detect related proteins. All data were expressed as the mean ± SD*, n* = 3. Student’s t-test was performed to analyze significance. *P < 0.05, **P < 0.01, ***P < 0.001.

GSEA analysis using the CGGA database showed that the first two most significantly enriched signaling pathways among the high OTUD4 gene expression phenotype are those associated with FGFR1 and oncogenic MAPK signaling pathways (Fig. 5A, Fig. 6A, Supplementary Fig. 6A). FGF/FGFR signaling is essential for growth and invasion of human GBM cells [22]. Databases also displays that FGFR1 is highly expressed in GBM and is positively associated with poor prognosis (Supplementary Fig. 6B, C). Interestingly, studies have shown that FGFR1 signaling pathway can stimulate CDK activity [23] and inhibit cyclin kinase inhibitors (CDKNs) [24]. As a result, we conducted further experiments to detect their relationship. Co-IP and PLA assays found that OTUD4 interacts with FGFR1(Fig. 5B, C, Supplementary Fig. 6D). And western blot showed that overexpression of OTUD4 stabilized whereas depletion of OTUD4 destabilized FGFR1, qRT-PCR assays confirmed that OTUD4 positively regulated FGFR1 at the protein level but not at the mRNA level (Supplementary Fig. 6E–G). Furthermore, MG132 significantly reduces the downregulation of FGFR1 caused by OTUD4 knockdown (Fig. 5D), suggesting that OTUD4 regulates FGFR1 in a manner related to the proteasome degradation pathway. CHX assay found that OTUD4 overexpression prolongs the half-life of FGFR1 (Fig. 5E, Supplementary Fig. 6H). We next sought to determine the linkage specificity of OTUD4-mediated deubiquitination of FGFR1. To this end, a specific deubiquitylation assay was adopted and exhibited that OTUD4 effectively identified and removed the K6 and K27-linked polyubiquitination of FGFR1 (Fig. 5F–I, Supplementary Fig. 6I). Next, our results corroborated that FGFR1 interference exacerbated the CDK1 downregulation caused by OTUD4 knockdown, and FGFR1 may maintain CDK1 protein stability by stabilizing the CDK1-cyclinB1 complex (Fig. 5J, K).

### The OTUD4-CDK1-MAPK axis is required in GBM progression

MAPK pathway is involved in the regulation of tumor cell proliferation and cell cycle, and also plays a mediating and signal amplification role in tumor invasion, including GBM [25]. Interestingly, both CDK1 and FGFR1 can activate the downstream MAPK signaling pathway [26–33]. Therefore, we speculated that OTUD4 might regulate CDK1 and the MAPK signaling pathway, ultimately affecting the progression of GBM. Next, we conducted western blot assays in GBM cells and found that OTUD4 knockdown or FGFR1 knockdown significantly downregulated the phosphorylation levels of proteins in the MAPK signaling pathway without affecting their total protein levels (Fig. 6B, C). While the downregulation was partially eliminated after CDK1 expression was restored (Fig. 6D). Furthermore, MTT assay, colony formation assay, transwell assay and western blot assay results exhibited that overexpression of CDK1 could partially restore the phenotypes of GBM cell proliferation and invasion inhibition caused by OTUD4 knockdown, and the expressions of cell cycle and EMT-related proteins also changed correspondingly (Fig. 6E–H). Hence, we conclude that OTUD4 does affect the activation of the MAPK signaling pathway through the regulation of CDK1, and promote the proliferation and invasion ability of GBM cells.

### OTUD4 contributes to the GBM progression in mice

**Fig. 7:**
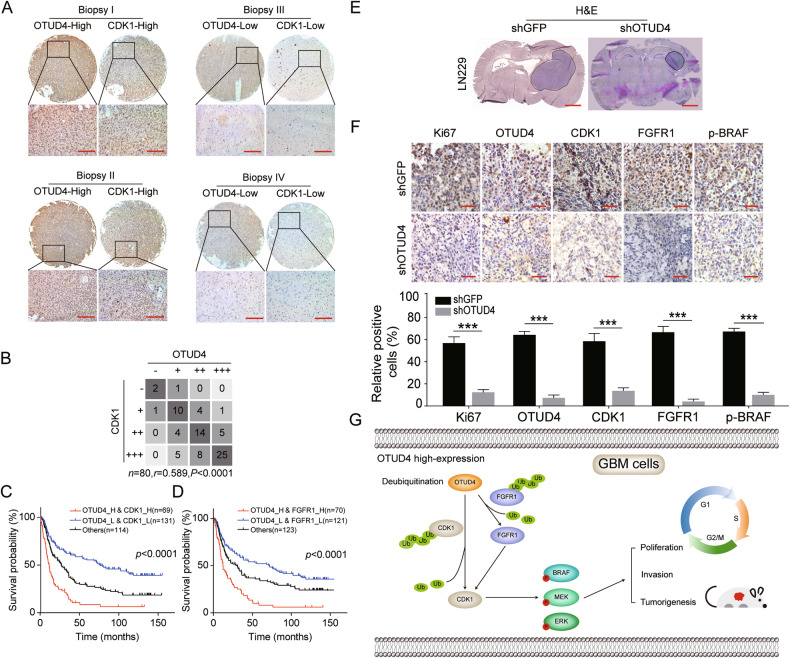
OTUD4 contributes to glioblastoma growth in vivo. **A** Representative IHC images with positive correlation between OTUD4 and CDK1 expression in human glioma tissue sample chips. Scale bar, 200 μm. **B** Spearman correlation test of OTUD4 and CDK1 expression. **C**, **D** Prognostic analysis using the CGGA database to explore the clinical correlation of OTUD4 and CDK1/FGFR1. **E** Representative hematoxylin-eosin (H&E) staining images of orthotopic implantation. Scale bar, 1 mm, *n* = 6. **F** Representative IHC images of the positive expression levels of Ki67(1:5000), OTUD4(1:300), CDK1(1:400), FGFR1(1:400) and p-BRAF(1:100). Scale bar, 50 μm. Data were expressed as the mean ± SD, *n* = 3. Student’s t-test was performed to analyze significance. *P < 0.05, **P < 0.01, ***P < 0.001. **G** Scheme for the regulatory mechanism of OTUD4 on CDK1.

Next, we aimed to explore the correlation between OTUD4 and CDK1 in clinical specimens. IHC assay found that samples with high OTUD4 expression were more likely to have high CDK1 staining intensity. In contrast, CDK1 staining was weak among OTUD4-low staining samples (Fig. 7A). Spearman correlation test further demonstrated the positive correlation between OTUD4 and CDK1 expression (Fig. 7B). Moreover, prognostic analysis using the CGGA database also revealed that patients had the worst prognosis when OTUD4 was highly expressed in combination with high CDK1 or FGFR1 expression (Fig. 7C, D). Together, there is an apparent clinical association of OTUD4-CDK1/FGFR1 in the human glioma development. To further evaluate the effect of OTUD4 on GBM cells proliferation in vivo, orthotopic implantation assay was performed in BALB/c-nu mice. The H&E results found that the tumor growth capability of GBM cells was significantly decreased after OTUD4 knockdown (Fig. 7E). IHC experiments displayed that the positive expressions of Ki67(a well-known marker of cell proliferation), OTUD4, CDK1, FGFR1 and p-BRAF were obviously decreased in tumor xenografts with OTUD4 knockdown (Fig. 7F). These results suggested that OTUD4 is required for GBM progression in vivo.

## Discussion

Glioblastoma is a common primary glioma known for its resistance to chemotherapy and radiation, its tendency to relapse frequently, and its poor prognosis [1]. It is urgent to find new drug targets for the treatment of GBM.

More and more evidence has proved that OTUs is closely related to the development of glioma. For example, OTUD3 promotes the progression of PTEN-associated glioma [34]. OTUB1 promotes the invasion of glioma cells, and inhibits CNS autoimmune [35, 36]. OTULIN maintains GSC self-renewal and is associated with poor prognosis in GBM [37]. As a member of the OTU family, OTUD4 has also been shown to affect cancer progression, for example, on the one hand, OTUD4 acts as an oncogene to facilitate the metastasis of triple-negative breast cancer [38] and melanoma [39]; on the other hand, OTUD4 acts as a tumor suppressor gene to inhibit the proliferation of liver cancer and non-small cell lung cancer, and regulate the radiosensitivity of nasopharyngeal carcinoma [40]. However, the effects of OTUD4 on GBM have not been reported. In this study, we report for the first time that OTUD4 may play an oncogenic role in GBM and may be considered as a potential prognostic biomarker for GBM. In addition, we found for the first time that OTUD4 directly or indirectly regulates the stability of CDK1 through deubiquitination, thus affecting the activation of the downstream MAPK signaling pathway, ultimately facilitating the GBM process (Fig. 7G).

It is generally accepted that OTU domain(1–180aa) is the main catalytic domain of OTUD4. However, studies reported that the deubiquitase recruitment domain (DRD, 181–550aa) of OTUD4 interacts with and deubiquitinates ALKBH3 [41, 42], intrinsically disordered regions (IDRs, such as 885–1114aa) of OTUD4 interacts with RNA [43], the ubiquitin interaction motif (UIM, 271–300aa) of OTUD4 promotes its DUB activity against MyD88 [10]. Similarly, in our study, 301–550aa of OTUD4 is the catalytic domain for deubiquitination of CDK1, and 181–300aa of OTUD4 might promote the DUB activity of OTUD4 on CDK1 by enhancing their interaction ability. These suggested that the domains that mediate the binding of OTUD4 to different substrates and the domains responsible for its DUB catalytic activity may be different.

Moreover, most human OTU enzymes are linkage specific, preferentially cleaving one, two, or a defined subset of linkage types. For example, OTUD4 has been reported to preferentially cleave K48-linked polyubiquitin chains [41, 44]. Phosphorylation of OTUD4 activates its K63-specific DUB activity [10]. Consistent with previous findings, our research also confirmed that K11-linked polyubiquitination is closely related to the cell cycle [45], and K11, K29-linked ubiquitination is essential for proteasomal degradation [46, 47]. In addition, we further speculated that K6-, K27-, and K33-linked ubiquitin chains may also be related to protein degradation.

Currently, CDK1 inhibitors for GBM have achieved certain effects [48, 49]. But deficiencies such as drug resistance have gradually emerged. Our findings established that OTUD4/CDK1 is clinically correlated and the OTUD4-CDK1-MAPK axis plays an important role in GBM progression. Considering that, our results help to identify new GBM-targeted inhibitor markers.

In conclusion, this study demonstrated that OTUD4 is an oncogene with prognostic significance in GBM, revealed a new mechanism by which OTUD4 directly deubiquitinates or indirectly regulates CDK1, and identified specific interaction regions and key amino acid residues of ubiquitin and CDK1(Fig. 7G). Accordingly, these results suggest that OTUD4 may serve as a biomarker for glioblastoma and provide a novel therapeutic target for GBM patients.

### Supplementary information

Supplementary Figures

aj-checklist

original western blot

## Data Availability

All data analyzed or generated in this study are included in this article as well as in the Supplementary Information file.

## References

[1] Louis DN, Perry A, Wesseling P, Brat DJ, Cree IA, Figarella-Branger D (2021). The 2021 WHO classification of tumors of the central nervous system: a summary. Neuro Oncol.

[2] Sahm F, Brandner S, Bertero L, Capper D, French PJ, Figarella-Branger D (2023). Molecular diagnostic tools for the World Health Organization (WHO) 2021 classification of gliomas, glioneuronal and neuronal tumors; an EANO guideline. Neuro Oncol.

[3] Wesseling P, Capper D (2018). WHO 2016 classification of gliomas. Neuropathol Appl Neurobiol.

[4] Miller KD, Ostrom QT, Kruchko C, Patil N, Tihan T, Cioffi G (2021). Brain and other central nervous system tumor statistics, 2021. CA Cancer J Clin.

[5] Ostrom QT, Patil N, Cioffi G, Waite K, Kruchko C, Barnholtz-Sloan JS (2020). CBTRUS statistical report: primary brain and other central nervous system tumors diagnosed in the United States in 2013–2017. Neuro Oncol.

[6] van den Bent MJ, Geurts M, French PJ, Smits M, Capper D, Bromberg JEC (2023). Primary brain tumours in adults. Lancet.

[7] Stupp R, Brada M, van den Bent MJ, Tonn JC, Pentheroudakis G (2014). High-grade glioma: ESMO Clinical Practice Guidelines for diagnosis, treatment and follow-up. Ann Oncol.

[8] Zhao X, Su X, Cao L, Xie T, Chen Q, Li J (2020). OTUD4: a potential prognosis biomarker for multiple human cancers. Cancer Manag Res.

[9] Wu Z, Qiu M, Guo Y, Zhao J, Liu Z, Wang H (2019). OTU deubiquitinase 4 is silenced and radiosensitizes non-small cell lung cancer cells via inhibiting DNA repair. Cancer Cell Int.

[10] Zhao Y, Mudge MC, Soll JM, Rodrigues RB, Byrum AK, Schwarzkopf EA (2018). OTUD4 is a phospho-activated K63 deubiquitinase that regulates MyD88-dependent signaling. Mol Cell.

[11] Zhang G, Tan R, Wan S, Yang R, Hu X, Zhao E (2022). HECTD3 regulates the tumourigenesis of glioblastoma by polyubiquitinating PARP1 and activating EGFR signalling pathway. Br J Cancer.

[12] Hou J, Xu M, Gu H, Pei D, Liu Y, Huang P (2022). ZC3H15 promotes glioblastoma progression through regulating EGFR stability. Cell Death Dis.

[13] Hu X, Liu R, Hou J, Peng W, Wan S, Xu M (2022). SMARCE1 promotes neuroblastoma tumorigenesis through assisting MYCN-mediated transcriptional activation. Oncogene.

[14] Li Y, Su Y, Zhao Y, Hu X, Zhao G, He J (2021). Demethylzeylasteral inhibits proliferation, migration, and invasion through FBXW7/c-Myc axis in gastric cancer. MedComm.

[15] Camacho-Urkaray E, Santos-Juanes J, Gutiérrez-Corres FB, García B, Quirós LM, Guerra-Merino I (2018). Establishing cut-off points with clinical relevance for bcl-2, cyclin D1, p16, p21, p27, p53, Sox11 and WT1 expression in glioblastoma - a short report. Cell Oncol.

[16] Han Z, Jia Q, Zhang J, Chen M, Wang L, Tong K (2023). Deubiquitylase YOD1 regulates CDK1 stability and drives triple-negative breast cancer tumorigenesis. J Exp Clin Cancer Res.

[17] Yan Y, Tao H, He J, Huang SY (2020). The HDOCK server for integrated protein-protein docking. Nat Protoc.

[18] Yan Y, Zhang D, Zhou P, Li B, Huang SY (2017). HDOCK: a web server for protein-protein and protein-DNA/RNA docking based on a hybrid strategy. Nucleic Acids Res.

[19] Yan Y, Wen Z, Wang X, Huang SY (2017). Addressing recent docking challenges: a hybrid strategy to integrate template-based and free protein-protein docking. Proteins.

[20] Huang SY, Zou X (2014). A knowledge-based scoring function for protein-RNA interactions derived from a statistical mechanics-based iterative method. Nucleic Acids Res.

[21] Huang SY, Zou X (2008). An iterative knowledge-based scoring function for protein-protein recognition. Proteins.

[22] Alshahrany N, Begum A, Siebzehnrubl D, Jimenez-Pascual A, Siebzehnrubl FA (2023). Spatial distribution and functional relevance of FGFR1 and FGFR2 expression for glioblastoma tumor invasion. Cancer Lett.

[23] Zhang Y, Lin Y, Bowles C, Wang F (2004). Direct cell cycle regulation by the fibroblast growth factor receptor (FGFR) kinase through phosphorylation-dependent release of Cks1 from FGFR substrate 2. J Biol Chem.

[24] Dombrowski C, Helledie T, Ling L, Grünert M, Canning CA, Jones CM (2013). FGFR1 signaling stimulates proliferation of human mesenchymal stem cells by inhibiting the cyclin-dependent kinase inhibitors p21(Waf1) and p27(Kip1). Stem Cells.

[25] Santarpia L, Lippman SM, El-Naggar AK (2012). Targeting the MAPK-RAS-RAF signaling pathway in cancer therapy. Expert Opin Ther Targets.

[26] Biswas PK, Kwak Y, Kim A, Seok J, Kwak HJ, Lee M (2022). TTYH3 modulates bladder cancer proliferation and metastasis via FGFR1/H-Ras/A-Raf/MEK/ERK pathway. Int J Mol Sci.

[27] Borysov SI, Cheng AW, Guadagno TM (2006). B-Raf is critical for MAPK activation during mitosis and is regulated in an M phase-dependent manner in Xenopus egg extracts. J Biol Chem.

[28] Borysov SI, Guadagno TM (2008). A novel role for Cdk1/cyclin B in regulating B-raf activation at mitosis. Mol Biol Cell.

[29] Cheng C, Wang J, Xu P, Zhang K, Xin Z, Zhao H (2022). Gremlin1 is a therapeutically targetable FGFR1 ligand that regulates lineage plasticity and castration resistance in prostate cancer. Nat Cancer.

[30] Kaibori Y, Katayama K, Tanaka Y, Ikeuchi M, Ogawa M, Ikeda Y (2020). Kinase activity-independent role of EphA2 in the regulation of M-phase progression. Exp Cell Res.

[31] Kaibori Y, Saito Y, Nakayama Y (2019). EphA2 phosphorylation at Ser897 by the Cdk1/MEK/ERK/RSK pathway regulates M-phase progression via maintenance of cortical rigidity. FASEB J.

[32] Liang G, Liu Z, Wu J, Cai Y, Li X (2012). Anticancer molecules targeting fibroblast growth factor receptors. Trends Pharmacol Sci.

[33] Yang J, Xu WW, Hong P, Ye F, Huang XH, Hu HF (2019). Adefovir dipivoxil sensitizes colon cancer cells to vemurafenib by disrupting the KCTD12-CDK1 interaction. Cancer Lett.

[34] Liu YZ, Du XX, Zhao QQ, Jiao Q, Jiang H (2020). The expression change of OTUD3-PTEN signaling axis in glioma cells. Ann Transl Med.

[35] Wang X, Mulas F, Yi W, Brunn A, Nishanth G, Just S (2019). OTUB1 inhibits CNS autoimmunity by preventing IFN-γ-induced hyperactivation of astrocytes. EMBO J.

[36] Xu L, Li J, Bao Z, Xu P, Chang H, Wu J (2017). Silencing of OTUB1 inhibits migration of human glioma cells in vitro. Neuropathology.

[37] Du X, Pang J, Gu B, Si T, Chang Y, Li T (2023). A bio-orthogonal linear ubiquitin probe identifies STAT3 as a direct substrate of OTULIN in glioblastoma. Nucleic Acids Res.

[38] Ma X, Wan R, Wen Y, Liu T, Song Y, Zhu Y (2024). Deubiquitinating enzyme OTUD4 regulates metastasis in triple-negative breast cancer by stabilizing Snail1. Exp Cell Res.

[39] Gao Y, Tang J, Ma X, Zhang C, Huang L, Che J (2023). OTUD4 regulates metastasis and chemoresistance in melanoma by stabilizing Snail1. J Cell Physiol.

[40] Di M, Miao J, Pan Q, Wu Z, Chen B, Wang M (2022). OTUD4-mediated GSDME deubiquitination enhances radiosensitivity in nasopharyngeal carcinoma by inducing pyroptosis. J Exp Clin Cancer Res.

[41] Zhao Y, Majid MC, Soll JM, Brickner JR, Dango S, Mosammaparast N (2015). Noncanonical regulation of alkylation damage resistance by the OTUD4 deubiquitinase. EMBO J.

[42] Liu R, Zhao E, Yu H, Yuan C, Abbas MN, Cui H (2023). Methylation across the central dogma in health and diseases: new therapeutic strategies. Signal Transduct Target Ther.

[43] Das R, Schwintzer L, Vinopal S, Aguado Roca E, Sylvester M, Oprisoreanu AM (2019). New roles for the de-ubiquitylating enzyme OTUD4 in an RNA-protein network and RNA granules. J Cell Sci.

[44] Mevissen TE, Hospenthal MK, Geurink PP, Elliott PR, Akutsu M, Arnaudo N (2013). OTU deubiquitinases reveal mechanisms of linkage specificity and enable ubiquitin chain restriction analysis. Cell.

[45] Jin L, Williamson A, Banerjee S, Philipp I, Rape M (2008). Mechanism of ubiquitin-chain formation by the human anaphase-promoting complex. Cell.

[46] Wu T, Merbl Y, Huo Y, Gallop JL, Tzur A, Kirschner MW (2010). UBE2S drives elongation of K11-linked ubiquitin chains by the anaphase-promoting complex. Proc Natl Acad Sci USA.

[47] Meyer HJ, Rape M (2014). Enhanced protein degradation by branched ubiquitin chains. Cell.

[48] Knockaert M, Greengard P, Meijer L (2002). Pharmacological inhibitors of cyclin-dependent kinases. Trends Pharmacol Sci.

[49] Goga A, Yang D, Tward AD, Morgan DO, Bishop JM (2007). Inhibition of CDK1 as a potential therapy for tumors over-expressing MYC. Nat Med.

