# Supplementary material for: OTUD4 promotes the progression of glioblastoma by deubiquitinating CDK1 and activating MAPK signaling pathway
Source: Cell Death Dis. 2024 Mar 1;15(3):179. doi: 10.1038/s41419-024-06569-x (PMC10907623; doi:10.1038/s41419-024-06569-x)
Supplement: Supplementary file 1 — Supplementary Figures [file 41419_2024_6569_MOESM1_ESM.docx]

**Supplementary Figure Legends**

**
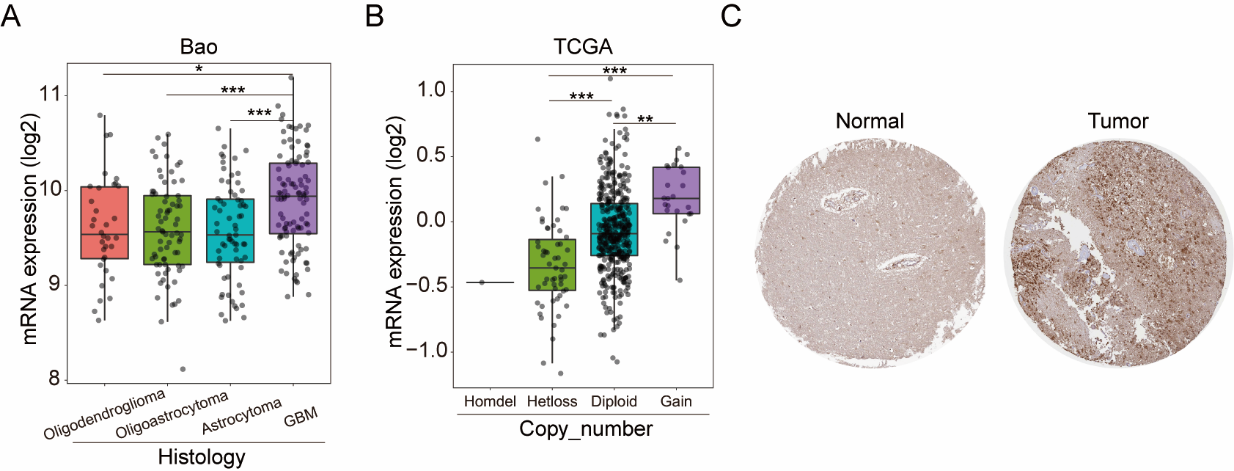
**

**Supplementary Figure. 1 OTUD4 is abnormally activated in GBM**

**(A, B)** Box plots of OTUD4 expression levels in Histology and Copy number glioma sets. **(C)** The Human Protein Atlas database was adopted to check expression of OTUD4 in human nontumor brain tissues and glioblastoma.


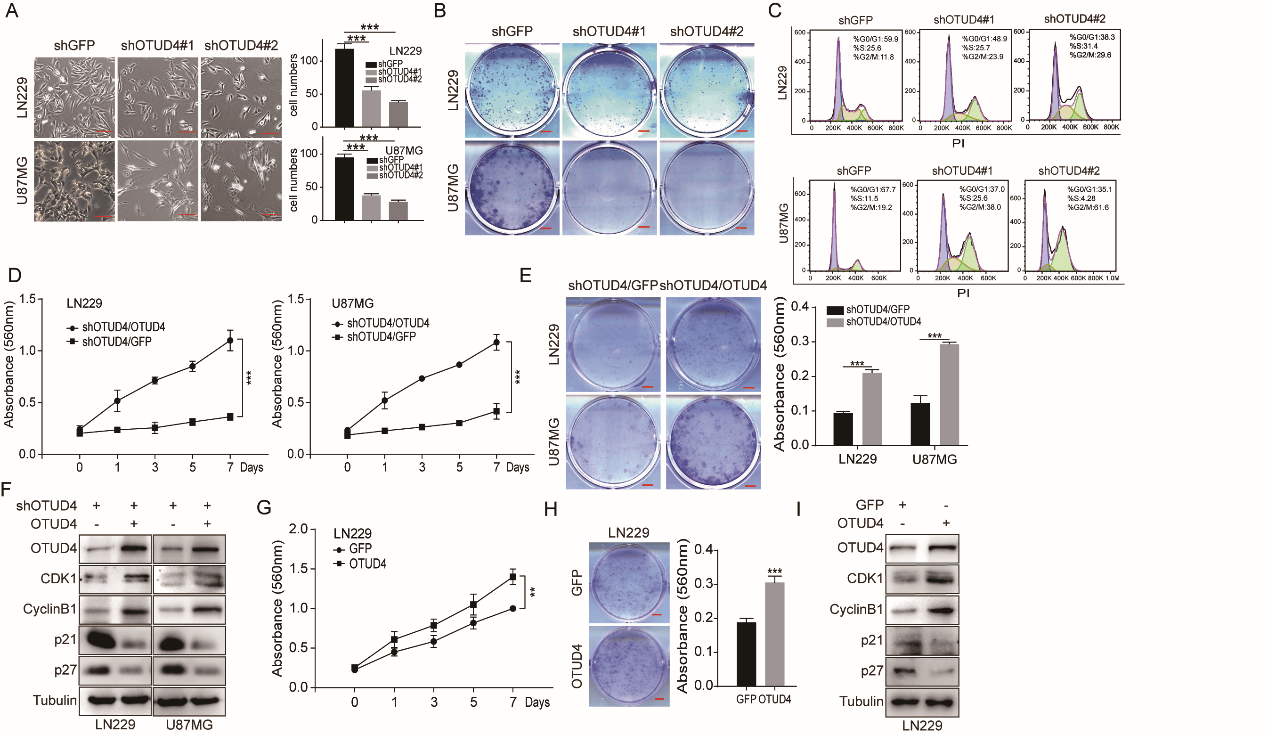


**Supplementary Figure. 2 OTUD4 is necessary for the proliferative ability of GBM cells**

**(A)** Morphology and numbers of cells were observed in the control and OTUD4-knockdown LN229 and U87MG cells. Scale bar, 20 μm. **(B, C)** Plate cloning assay and flow cytometry were performed to test the effect of OTUD4 knockdown on cell proliferation. Scale bar, 2 mm. **(D-F)** The effect of OTUD4 overexpression in OTUD4-knockdown GBM cells on proliferation was detected by MTT, plate cloning and western blot assay. Scale bar, 2 mm. **(G-I)** MTT assay, colony formation assay and western blot assay were repeated to detect cell proliferation capacity and related protein expression levels in LN229 cells overexpressing OTUD4. Scale bar, 2 mm.All data were expressed as the mean ± SD, *n*=3. Student’s t test was performed to analyze significance. **P* < 0.05, ***P* < 0.01, ****P* < 0.001.


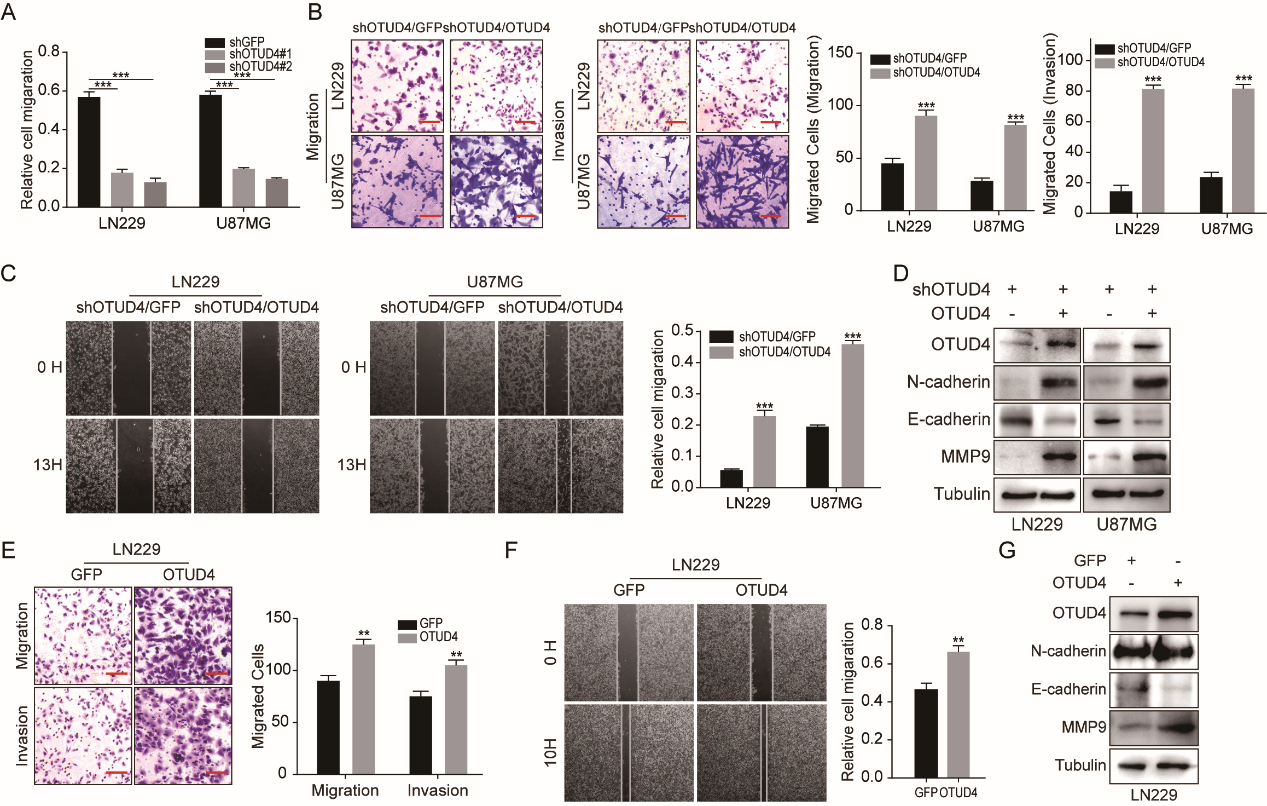


**Supplementary Figure. 3 OTUD4 is necessary for the invasion ability of GBM cells**

**(A)** Wound-healing assay was performed in the control and OTUD4-knockdown cells. **(B-D)** The effect of OTUD4 overexpression in OTUD4-knockdown GBM cells on invasion was detected by transwell assay, wound-healing assay and western blot assay. Scale bar, 20 μm. **(E-G)** Transwell assay, wound-healing assay and western blot assay were carried out in OTUD4-overexpression LN229 cell. Scale bar, 20 μm. All data were expressed as the mean ± SD, *n*=3. Student’s t test was performed to analyze significance. **P* < 0.05, ***P* < 0.01, ****P* < 0.001.


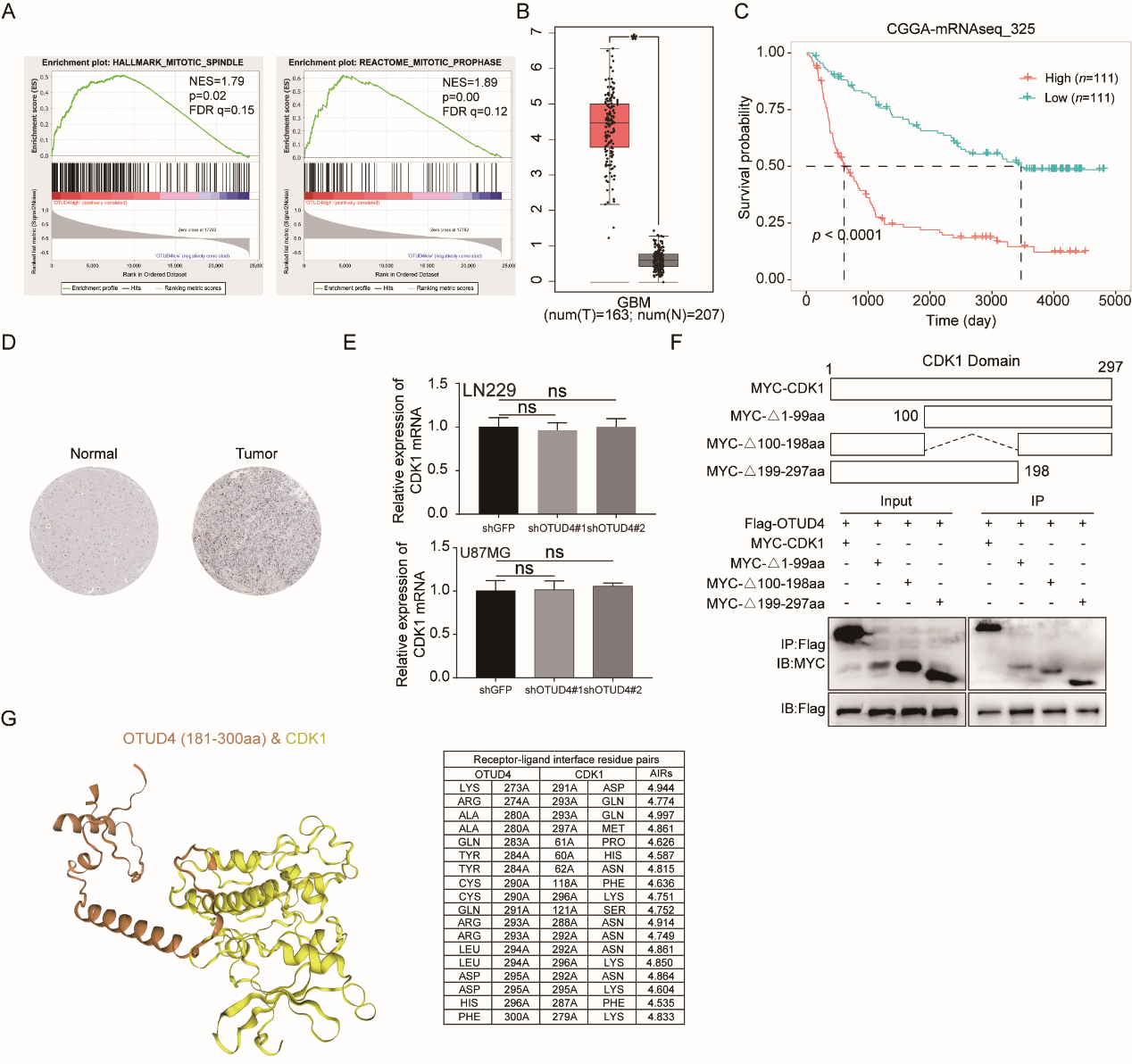


**Supplementary Figure. 4 OTUD4 interacts with CDK1**

**(A)** GSEA plots depicted enrichment of the mitotic spindle and mitotic prophase in the samples with high OTUD4 expression. FDR, false discovery rate; NES, normalized enrichment score. **(B)** GEPIA database showed that CDK1 is highly expressed in GBM. **(C)** Kaplan-Meier analysis using CGGA database to reveal the prognostic importance of CDK1 in GBM. **(D)** The expression of CDK1 in human nontumor brain tissues and glioblastoma. **(E)** Changes of CDK1 mRNA levels in GBM cells after OTUD4 knockdown. **(F)** Interaction detection between OTUD4 and CDK1 truncated domains in 293FT cells. **(G)** SWISS-MODEL database and HADDOCK database were used to simulate the structure of OTUD4 (181-300aa) and CDK1, and perform molecular docking (Docking Score: -240.05, Coincidence Score: 0.8583). Possible interface residue pairs and corresponding Ambiguous Interaction Restraints (AIRs) were listed. All data were expressed as the mean ± SD, *n*=3. Student’s t test was performed to analyze significance. **P* < 0.05, ***P* < 0.01, ****P* < 0.001.


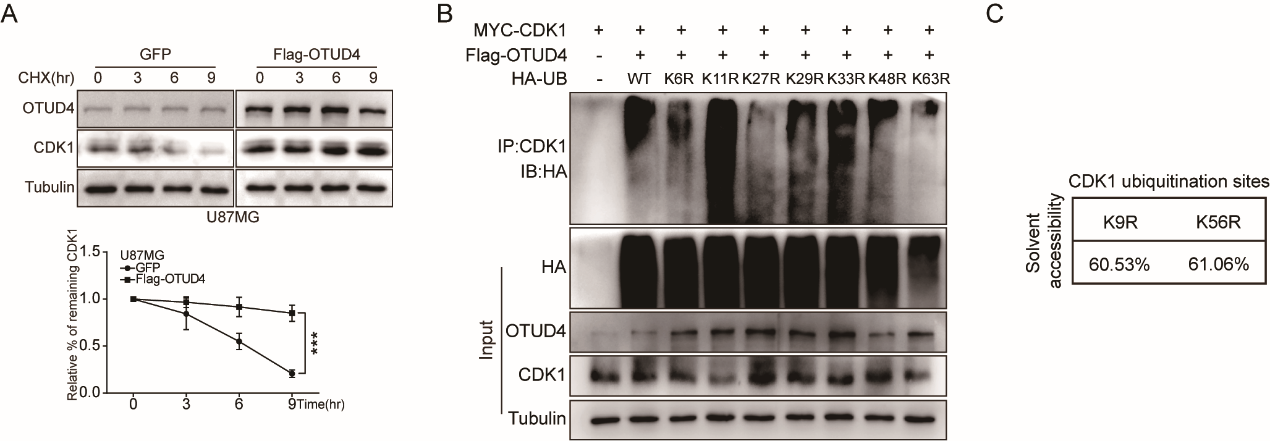


**Supplementary Figure. 5 OTUD4 stabilizes CDK1 by deubiquitination**

**(A)** Western blot analysis of the turnover of CDK1. **(B)** In the presence of MG132, the Flag-OTUD4, MYC-CDK1, HA-UB, and ubiquitin mutant plasmids (only one lysine residue was mutated to an arginine residue) were co-transfected into 293FT cells for ubiquitination assays. **(C)** Schematic representation of the solvent accessibility of CDK1 ubiquitination sites. All data were expressed as the mean ± SD, *n*=3. Student’s t test was performed to analyze significance. **P* < 0.05, ***P* < 0.01, ****P* < 0.001.


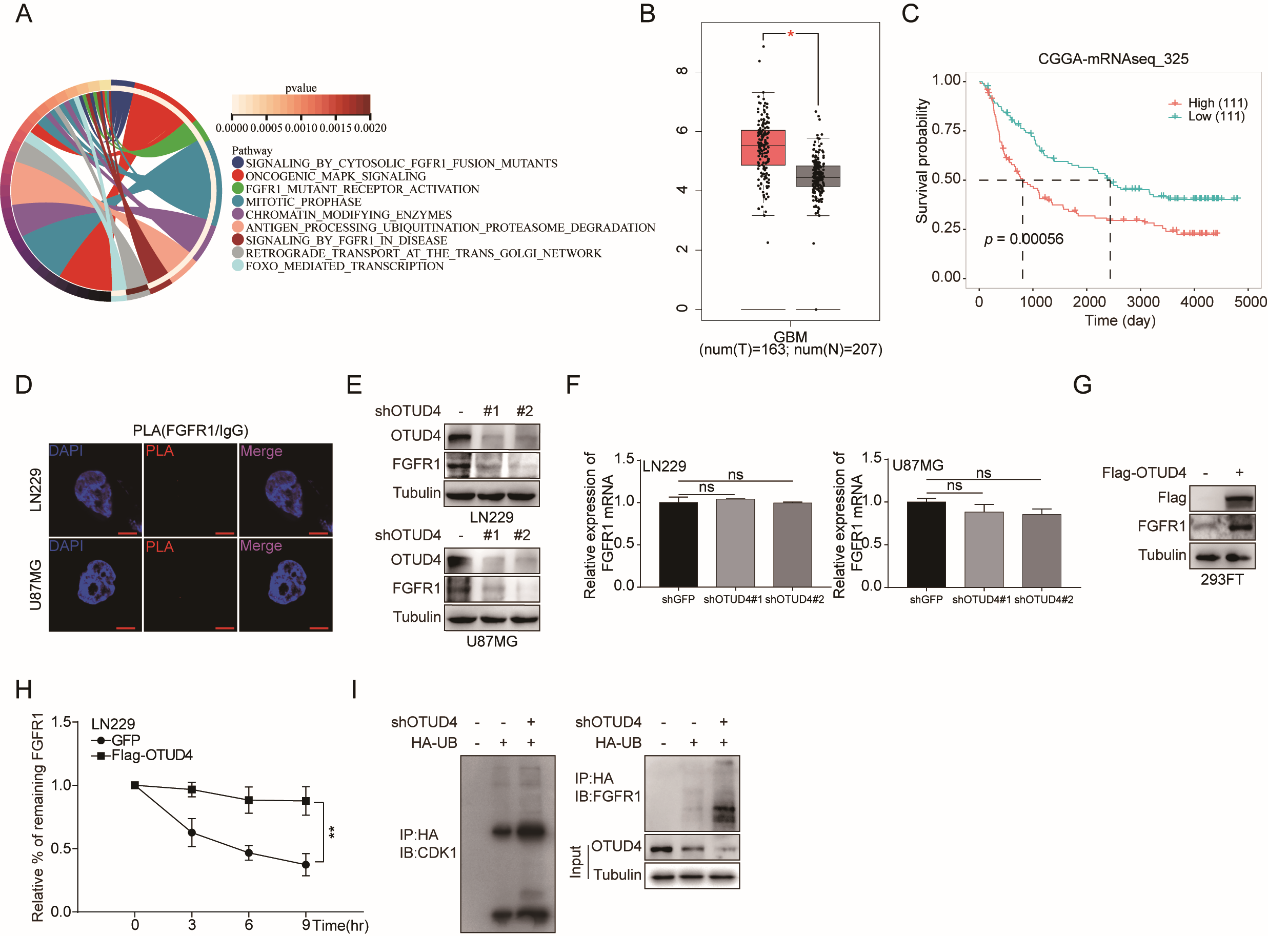


**Supplementary Figure. 6 OTUD4 interacts with FGFR1 and stabilizes FGFR1 by deubiquitination**

**(A)** Signaling pathways enrichment plots in GBM cases with high OTUD4 expression. **(B)** GEPIA database showed that FGFR1 is highly expressed in GBM. **(C)** Kaplan-Meier analysis using CGGA database to reveal the prognostic importance of FGFR1 in GBM. **(D)** The proximity ligation (PLA) assay was applied to detect the interaction of FGFR1 and IgG in GBM cells. Scale bar, 10 μm. **(E-G)** Changes of FGFR1 protein and mRNA levels in GBM cells after OTUD4 knockdown or overexpression. **(H)** Quantitative analysis of the turnover of FGFR1. **(I)** In the presence of MG132, 293FT cells were co-transfected with shOTUD4 and HA-UB plasmids, and the ubiquitinated CDK1 and FGFR1 were detected by co-IP. All data were expressed as the mean ± SD, *n*=3. Student’s t test was performed to analyze significance. **P* < 0.05, ***P* < 0.01, ****P* < 0.001.
