# Supplementary figures and images for: OTUD4 promotes the progression of glioblastoma by deubiquitinating CDK1 and activating MAPK signaling pathway
Source: Cell Death Dis. 2024 Mar 1;15(3):179. doi: 10.1038/s41419-024-06569-x (PMC10907623; doi:10.1038/s41419-024-06569-x)

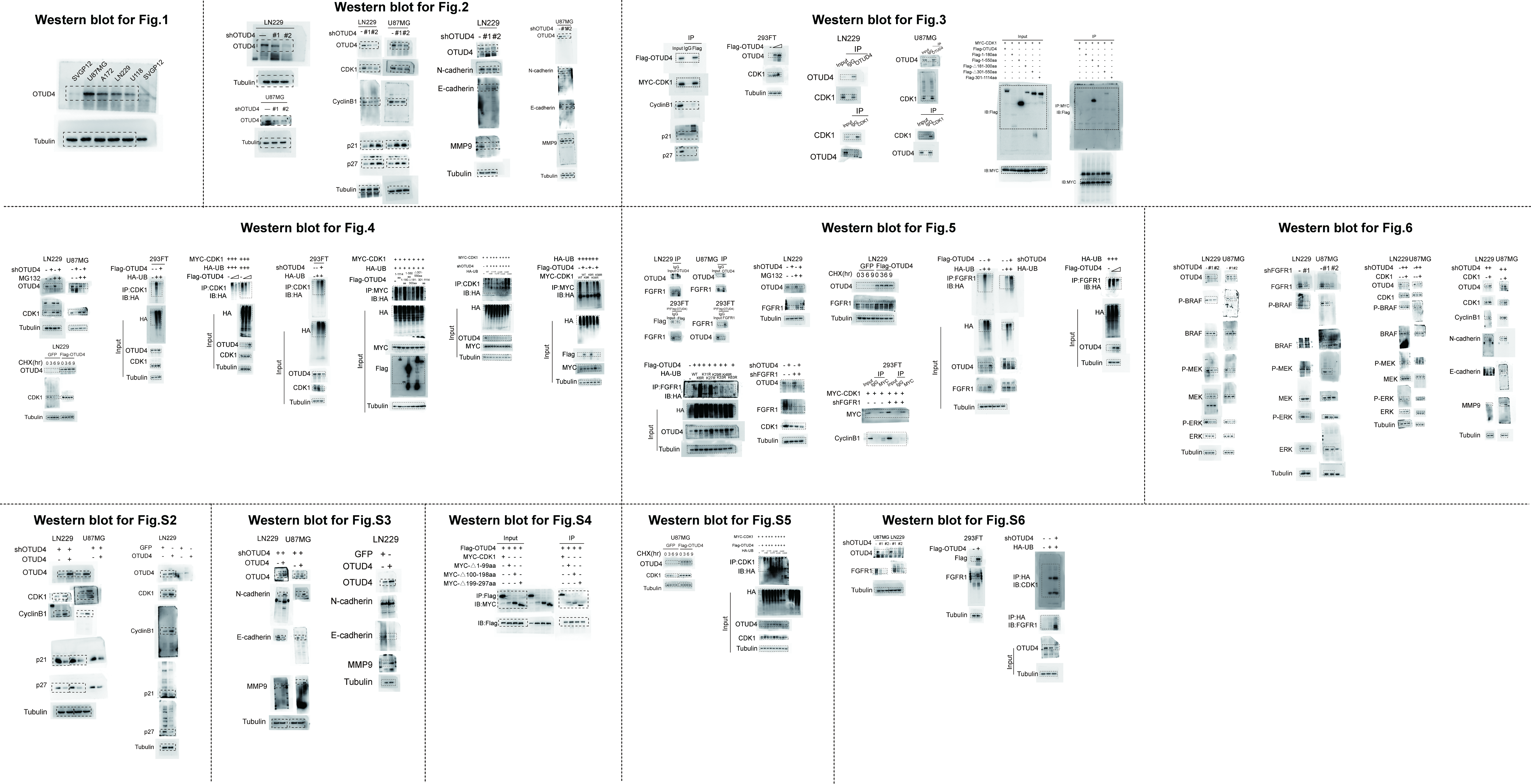

Supplement: Supplementary file 3 — original western blot [file 41419_2024_6569_MOESM3_ESM.tif]
